# Patients With Chronic Three-Vessel Disease in a 15-Year Follow-Up Study: Genetic and Non-Genetic Predictors of Survival

**DOI:** 10.1097/MD.0000000000000278

**Published:** 2014-12-02

**Authors:** Jan Máchal, Monika Pávková-Goldbergová, Ota Hlinomaz, Ladislav Groch, Anna Vašků

**Affiliations:** From the Department of Pathophysiology, Faculty of Medicine, Masaryk University Brno, Czech Republic (JM, MPG, AV); International Clinical Research Center, St. Anne's University Hospital, Brno, Czech Republic (JM, LG, OH); and First Department of Internal Medicine – Cardioangiology, St. Anne's University Hospital, Brno, Czech Republic (OH, LG).

## Abstract

Genetic and non-genetic predictors of 15-year survival in patients with chronic three-vessel disease (3VD) were investigated.

Coronary angiography was performed on 810 subjects with symptoms of stable ischemic heart disease in 1998. The patients with 3VD were genotyped for 23 candidate polymorphisms covering the PPAR-RXR pathway, matrix metalloproteinase-2, renin–angiotensin–aldosterone system, endothelin-1, cytokine genes, MTHFR and APO E variants. Fifteen-year survival data were obtained from the national insurance registry. All data were available in the case of 150 patients with 3VD. Statistical analysis used stepwise Cox regression with dominant, recessive, or additive mode of genetic expression. Involved variables included age, sex, BMI, blood pressure, diabetes, ejection fraction, left main stenosis, previously diagnosed coronary stenosis, myocardial infarction in personal history, and coronary bypass along with polymorphisms pre-selected by log-rank tests.

Out of the 23 polymorphisms, four were included in the model construction. SNP in the IL-6 gene rs1800795 (−174 G/C) has been found to be a significant predictor of survival. This SNP was in a linkage disequilibrium with rs1800797 (−597 G/A) in the same gene (D′ = 1.0), which was also found to constitute a significant predictor of survival when rs1800795 was not included in the model construction. Age, increased BMI, diabetes, low EF, and left main stenosis were also significant predictors in all models.

Age, increased BMI, diabetes, low ejection fraction, left main stenosis, and genetic variation in the IL-6 promoter were established as significant independent risk factors for the survival of patients with three-vessel disease.

## INTRODUCTION

Coronary artery disease (CAD) and its complications – such as myocardial infarction or heart failure – is one of the leading causes of death in most world populations.^1,2^ Three-vessel disease (3VD) represents the most severe form of coronary atherosclerosis. Patients with 3VD and/or left main stenosis are considered a high-risk group according to therapeutic guidelines.^3^ In comparison with less severe forms of CAD, 3VD has been consistently associated with worse long-term prognosis.^4–7^ Genetic variation in several metabolic, inflammatory, and local signal pathways is worthy of consideration concerning possible effects on patients’ survival.

Overall heritability of CAD has been estimated at approximately 50% in population studies and over 30 genes have been associated with CAD onset in genome-wide association studies (GWAS).^8^ While the genome-wide association approach explains only a small fraction of total heritability,^9^ candidate gene-based studies often suffer from various types of bias which may lead to both false positive and false negative results.^10–12^ The role of various suspected genetic risk factors in the survival of patients already suffering from symptomatic CAD is not yet well understood.

As atherosclerosis is an inflammatory process, cytokines play an important role in its pathogenesis. Cytokines such as tumour necrosis factor (TNF) α and interleukin (IL) 6 have been extensively studied. While TNF-α seems to be clearly pro-atherogenic, the role of IL-6 is somewhat ambivalent in animal and human studies.^13^ The precursor of TNF-α is converted to its active form by its converting enzyme, TACE.^14^ TNF-β, also known as lymphotoxin α, is secreted by regulatory T-lymphocytes and exhibits anti-atherogenic effects.^15^

Regarding the variation of lipid metabolism pathways, one of the key molecules is apolipoprotein E (ApoE = protein, APO E = gene), a protein which ensures lipoprotein clearance, prevents lipid accumulation in the vessel wall,^16^ and has antioxidant,^17^ vasodilatory^18^ and anti-inflammatory^19^ effects. The peroxisome proliferator-activated receptor/retinoid X receptor (PPAR-RXR) pathway is involved in both the regulation of the lipid and glucose metabolism and in cytokine release.^20^ The lower expression of PPAR-γ and RXR-α has been associated with the faster progression of carotic atherosclerosis.^21^ Similarly, PPAR-α also has anti-atherogenic properties^22^

Contributing to local inflammation, matrix metalloproteinases (MMPs) are endopeptidases which degrade the extracellular matrix. Many MMPs are expressed in atherosclerotic vessels.^23,24^ Of these, MMP-2 has been found to participate in lesion formation in the animal model of atherosclerosis^25^ and its gene expression is higher in acute coronary syndrome patients compared to healthy subjects.^26^

The renin–angiotensin–aldosterone system (RAAS) plays also role in tissue remodelling and is an important regulator of blood pressure. The hyperactivity of RAAS is linked to cardiovascular diseases including hypertension and CAD. Angiotensin converting enzyme (ACE) is a key molecule activating angiotensin II, which is a strong vasoconstrictor.^27^ Endothelins are a group of other vasoconstriction peptides. Endothelin-1 (ET-1) is synthesized mostly in the vessel wall and is the most potent vasoconstrictor. Moreover, it exerts several other biological functions leading to elevated blood pressure.^28^

From other possible risk-modifying factors, methylene tetrahydrofolate reductase (MTHFR) is an enzyme important for homocysteine degradation. The overaccumulation of homocysteine is associated with higher risk of atherosclerosis, probably through various mechanisms.^29^

We conducted the study to establish the genetic and other factors contributing to all-cause death of patients with chronic symptomatic 3VD. The aim was to create a model predicting patients’ survival based on significant and independent risk factors. The non-genetic determinants that were considered to possibly play an important role in survival included the following clinical factors and characteristics of cardiac involvement: age at admission, sex, body mass index (BMI), systolic and diastolic blood pressure (SBP and DBP, respectively), diabetes mellitus (DM), hyperlipidemia, ejection fraction (EF), left main stenosis, extent of CAD, previously diagnosed stenosis of coronary artery, myocardial infarction in personal history, and mode of intervention – coronary artery bypass grafting (CABG), percutaneous coronary intervention (PCI), or pharmacological therapy. Potential genetic factors involved 23 candidate polymorphisms including the variants in genes coding RXR-a, RXR-b, PPAR-a, PPAR-g, endothelin-1, TNF-a, TACE, TNF-b, IL-6, MMP-2, angiotensinogen (AGT), ACE, MTHFR, and ApoE.

## METHODS

### Coronary Angiography and Patient Selection

Left and right coronary angiography and left ventriculography were performed on 810 consecutive subjects at the First Department of Internal Medicine – Cardioangiology at St. Anne's University Hospital in Brno in 1998. The subjects suffered from chest pain or other symptoms of stable ischemic heart disease. Coronary angiograms were assessed by four experienced invasive cardiologists. Of the total number of subjects, 196 suffered from 3VD, defined as ≥50% stenosis of the left anterior descendent branch (LAD), left circumflex branch (LCx), right coronary artery (RCA), and/or major branches of each artery. Extent of CAD was defined as the number of segments with ≥50% stenosis, according to the 16-segment scheme of American Heart Association.

Informed consent was obtained from all patients prior to their recruitment according to the requirements of Ethics committee of St. Anne's University Hospital, which approved the study. All the procedures were in accord with the Helsinki Declaration of 1975 as revised in 1983. Patients treated for concomitant significant valvular disease and those after heart transplantation were excluded. The remaining subjects were genotyped for 23 candidate polymorphisms and other clinical and laboratory data were collected. Data about 15-year survival were obtained from national insurance registry on May 23, 2013. All data were available in the case of 150 patients; only these subjects were included in subsequent analyses. The reasons of the exclusion of remaining 46 patients were incomplete genetic analysis of 23 polymorphisms (n = 40), incomplete clinical data (n = 5), and inability to obtain the data about patients survival from national insurance registry (n = 1). There was no difference in survival between patients included and excluded in the model (in case when survival data were available; Gehan Wilcoxon test *P*-value = .28).

### Laboratory Methods

DNA was extracted from peripheral blood leukocytes using the phenol–chloroform method. Of the total number of 23 polymorphisms, 21 were identified using polymerase chain reaction (PCR), and restriction analysis. For the single nucleotide polymorphism (SNP) rs1536475 (intron 7, +70 A/G) in the RXR-α gene, PCR was carried out in a volume of 25 μl, containing 0.8 U of Taq polymerase and primers 5-AGACAGCTGAGTGACTGTGTG C-3 (forward) and 5-GAAATAATACTAGGCAGGATGTGC-3 (reverse). The method was described in^30^ and the resulting fragment was 269 base pairs (bp) in length. The process of restriction analysis was modified in our laboratory and included digestion by SatI enzyme in 37°C and electrophoresis in 2% agarose gel (Serva). The resulting fragments were 162 + 66 + 41 bp (A allele) or 123 + 66 + 41 + 39 bp (G allele) in length.

The parameters of methods used in the detection of other polymorphisms have been described in our previous publications. This includes polymorphisms rs148360070 (intron 5 39526 A/AA) and rs1805343 (intron 9 −25 G/A) in the RXR-α gene and all variants in genes coding RXR-β,^31^ PPAR-α, PPAR-γ,^32^ endothelin-1, TNF-α, TACE, TNF-β,^33^ IL-6,^34,35^ MMP-2,^36^ angiotensinogen,^37^ ACE,^38^ and MTHFR.^39^

Two polymorphisms in the Apo E were determined using real-time PCR monitored by SYBR Green.^40^ The method was optimized by our research group.^41^

### Statistical Analysis

The Cox regression model was used in order to estimate the contribution of genetic polymorphisms and other risk factors to overall survival. The genetic variants had been pre-selected out of the total number of 23 polymorphisms in candidate genes using the Kaplan–Meier method and log-rank tests in dominant, recessive and co-dominant modes of allelic expression. A *P*-value of .1 was used as the cut-off for including the variable in the Cox regression analysis, the power of log-rank tests were 0.2 to 0.8 for each separate test, depending on allele and genotype frequency. Tests with lower power due to low number in one of the compared groups were not performed; the combination of three tests for each polymorphism increased power to at least 0.6 with the exception of APO E2. The Hardy–Weinberg equilibrium was calculated for each polymorphism using the χ^2^-test. To address potential selection bias, the log-rank tests were repeated for the variants included in the Cox regression model construction in all patients with 3VD, where the data about given variant and survival were available (150 < n < 196).

A stepwise construction of the Cox regression model with a *P*-value to include of .05 and *P*-value to remove of .051 was subsequently employed to determine the contribution of pre-selected genetic and other variables to overall survival. Non-genetic variables included in the stepwise construction were age at admission, sex, BMI, hyperlipidemia, systolic and diastolic blood pressure, DM, EF, left main stenosis, extent of CAD, previously diagnosed stenosis of coronary artery, myocardial infarction in personal history, and mode of treatment.

With the unknown allelic coefficient of dominance, three models of gene expressions were employed: dominant, where minor allele carriers were compared with major allele homozygotes, recessive, where major allele carriers were compared with minor allele homozygotes (calculated only when the number of minor allele homozygotes was more than five) and additive, a gene dose-based model where the values 0, 1 and 2 were attributed to major allele homozygotes, heterozygotes, and minor allele homozygotes.

Finally, an all-effects multivariate Cox model was used to determine the hazard ratio (HR) with 95% confidence interval (CI) of different genotypes of the SNPs determined as a significant factor in overall survival following adjustments for age, sex, body mass index (BMI), systolic and diastolic blood pressure (SBP and DBP, respectively), diabetes mellitus (DM), hyperlipidemia, left main stenosis, EF, extent of CAD, previously diagnosed stenosis of coronary artery, myocardial infarction in personal history, and mode of treatment. Bonferroni correction was used for multiple comparisons testing, the respective *P*-values are listed as p_corr_. Comparisons of continuous variables between specified groups of patients were performed using Mann–Whitney *U*-test; for categorical variables Fisher exact test was used. Generally, α = 0.05 was used to determine statistically significant results in all analyses. STATISTICA software (StatSoft, version 12) was used for statistical analysis. MIDAS software (version 1.0)^42^ was used for linkage disequilibrium determination.

## RESULTS

The basic characteristics of the group and its medication are shown in a table (Table 1). Approximately 50% of the patients with three-vessel disease died before the end of the study. The 5-year survival rate was 88%.

**TABLE 1:**
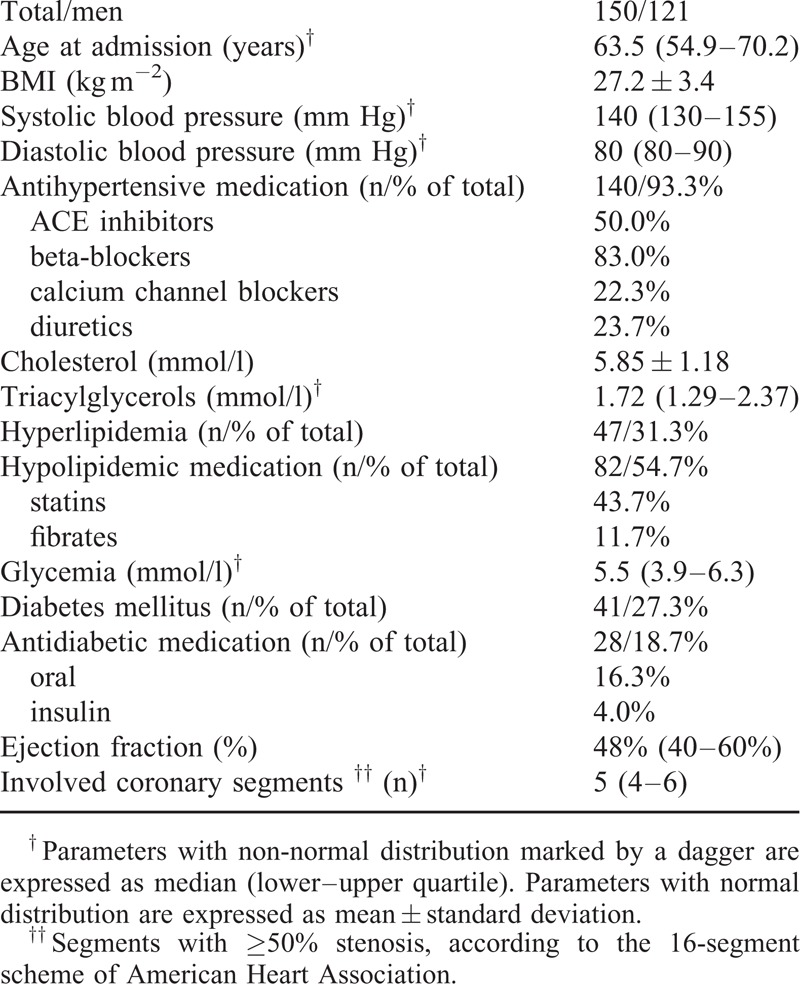
Basic Characteristics of the Subjects

The pre-selection of polymorphisms included in final model construction was based on log-rank *P*-values. Only the genetic variants with *P* < .10 in at least one model were selected for further analysis (Table 2). Polymorphisms rs1536475 in RXR-α, 1800629 in TNF-α, and two SNPs in Il-6 (rs1800795 and 1800797) met the inclusion criteria. When the log-rank tests were repeated in all patients with 3VD where the data about given variant and survival were available, only the two latter polymorphisms showed the *P*-value <.10 (and <.05, actually).

**TABLE 2:**
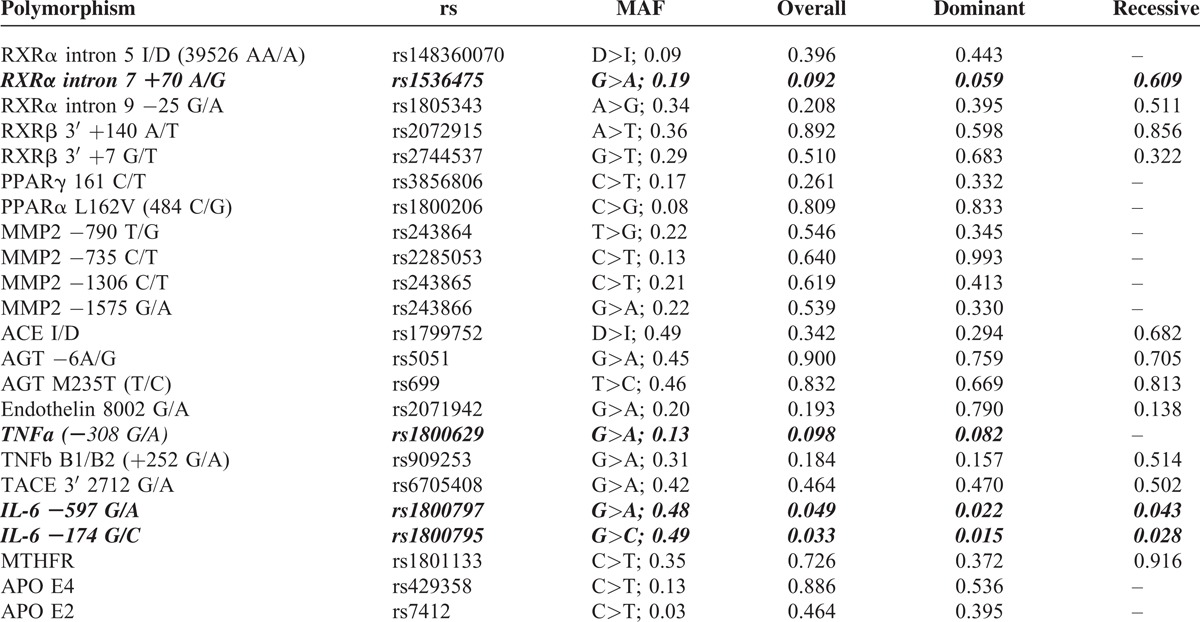
Candidate Polymorphisms, Their Respective Minor Allele Frequencies (MAF) and Log-Rank *P*-Values. Polymorphisms Included in the Stepwise Cox Regression Model Construction are Indicated in ***BoldItalics***

The stepwise Cox regression model construction identified the same significant predictors of survival consistently in all three models: age at admission, ejection fraction, left main stenosis, BMI, diabetes, and polymorphism rs1800795 (−174 G/C) in the IL-6 gene. In additive model, the effect of rs1800795 alleles remained significant after Bonferroni correction, the C allele was associated with worse prognosis. This SNP was in strong linkage disequilibrium with rs1800797 (−597 G/A) in the same gene (D′ = 1.0; *r*^2^ = .97; *P* < 10^−20^). The G–G haplotype was the most common (51%), followed by A–C (48%) and G–C (1%); A–G was missing in our group of patients. SNP rs1800797 was also a significant predictor of survival in the additive model when rs1800795 was not included in model construction. The G allele was protective and the A allele risky in this case. No other polymorphism was a significant independent predictor of survival (Table 3).

**TABLE 3:**
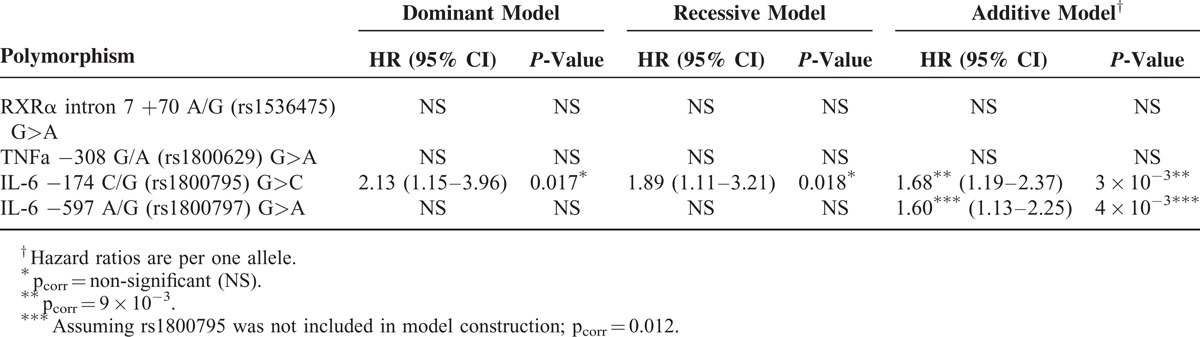
Effect of Selected Polymorphisms in Multivariate Analysis. *P*-Values and Hazard Ratios (HR) are Adjusted for Other Significant Independent Predictors of Survival. The Major Allele Was Taken as the Reference Variant

The final multivariate model obtained by stepwise Cox regression and including all significant predictors (with the additive effect of rs1800795 alleles) is shown in a table (Table 4).

**TABLE 4:**
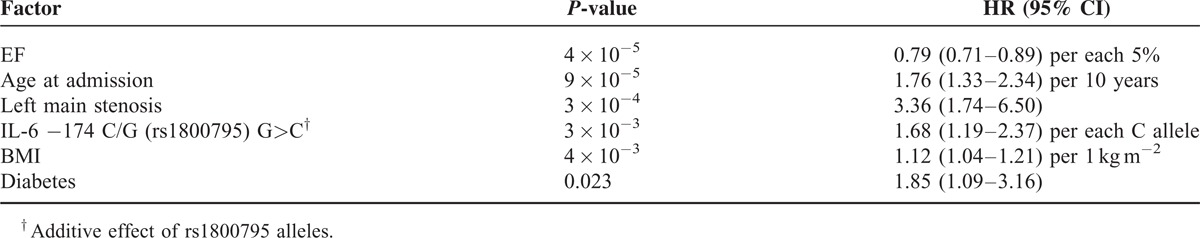
Final Multivariate Cox Regression Model Including All Significant Independent Predictors of Survival

**FIGURE 1:**
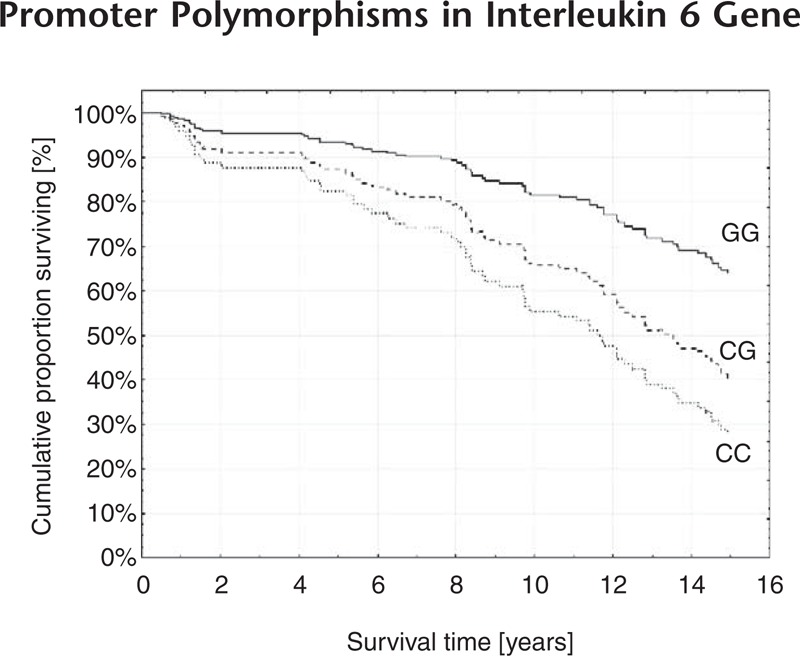
Effect of rs1800795 (−174 G/C) in the IL-6 gene on survival in multivariate analysis. Kaplan–Meier curves are adjusted for age, sex, diabetes, BMI, EF, SBP, DBP, left main stenosis, previously diagnosed coronary stenosis and myocardial infarction in personal history. Compared to GG homozygotes, both CG (HR = 2.19; 95% CI = 1.05–4.58; *P* = .04) and CC (HR = 3.79; 95% CI = 1.78–8.10; *P* = 6 × 10^−4^) carriers showed higher mortality.

Following adjustments for all clinical covariates (all effects model) and compared to GG carriers of rs1800795, CG carriers had a HR of 2.19 (95% CI = 1.05–4.58) and CC homozygotes had a HR of 3.79 (95% CI = 1.78–8.10). The Kaplan–Meier survival curves of different genotypes of rs1800795 following adjustment for age, sex, diabetes, BMI, EF, extent of CAD, dyslipidemia, SBP, DBP, left main stenosis, previously diagnosed coronary stenosis, myocardial infarction in personal history, and mode of intervention are shown in a graph (Figure 1). Analogically, compared to GG homozygotes in rs1800797, AG heterozygotes had a HR of 2.09 (95% CI = 1.01–4.33) and AA homozygotes exhibited a HR of 3.40 (95% CI = 1.61–7.16); a *P*-value was <.05 in all the cases.

Of the total number of 150 patients, a majority (n = 94) was treated by CABG, 28 patients underwent PCI, and the remaining subjects (n = 28) were treated pharmacologically. The CABG procedure was not a significant independent predictor of survival in the stepwise Cox regression model. In a separate analysis, CABG was superior to pharmacological therapy (log-rank test: *P* = .04); however, this effect became insignificant in a multivariate model (*P* = .18, HR = 0.65, 95% CI = 0.35–1.21) following adjustment for all clinical covariates. Patients treated by CABG had higher EF compared to patients treated by PCI or pharmacological therapy (*P* = 6 × 10^−3^), they did not differ in other investigated clinical parameters and characteristics of cardiac involvement.

## DISCUSSION

### Clinical and Angiographic Predictors of Mortality

This study revealed several significant independent predictors of mortality in patients with chronic 3VD. Characteristics of cardiac involvement, detected using coronary angiography, and ventriculography were important predictors of all-cause death. Specifically, left main stenosis increased the risk of death more than three-fold while each 5% of ejection fraction lowered the risk by approximately 20%. Other significant predictors of death – age, diabetes, and BMI – are considered to be established cardiovascular risk factors.^43–45^ Diabetes mellitus leads to hyperglycaemia which promotes the progression of atherosclerosis both directly, by non-enzymatic glycation of endothelial proteins,^46^ and indirectly, by lipoprotein modification and changes in their function.^47–49^

In this study, higher BMI was associated with a worse prognosis in multivariate analysis: an increase of 1 kg m^−2^ increased risk of death by over 12%. This result confirms the role of overweight as an independent risk factor in patients suffering from 3VD and is in contradiction with some studies and meta-analyses reporting on the so-called “obesity paradox,” that is, better prognosis in overweight cardiac patients.^50,51^ However, selection bias is most likely at least a component factor contributing to the obesity paradox.^52^ It must be noted that the study group assembled for the purposes of our study was relatively homogenous and that patients suffered from a severe form of coronary atherosclerosis. Prospective design, as used in our study, should reduce the possibility of selection bias which might influence the results of retrospective studies.

### Promoter Polymorphisms in Interleukin 6 Gene

Out of the 23 potential genetic predictors (including genetic variants of the PPAR-RXR pathway, matrix metalloproteinase-2, renin–angiotensin–aldosterone system, endothelin-1, cytokine genes, MTHFR and APO E), only two closely linked polymorphisms in the IL-6 promoter proved to have both statistical and clinical significance for patient survival. The role of IL-6 and its genetic variants in atherosclerosis is not fully clarified and results of many in vitro, animal model and human studies are controversial. In vitro, IL-6 stimulates angiogenesis^53^ while animal models of atherosclerosis have shown IL-6 to be both anti-atherosclerotic^54,55^ and pro-atherosclerotic,^56^ depending on concentration. It has also been found to have pro-coagulation and pro-inflammatory effects^57,58^ and to contribute to heart remodelling after myocardial infarction in human patients.^59^ The long-term elevation of IL-6 levels has been associated with the risk of CAD^60^ and CAD severity.^61^ Elevated concentrations of IL-6 are also predictors of mortality in patients with CAD or heart failure,^62,63^ although the causality is uncertain and the concentration of IL-6 largely varies in the same individual.^64^ “Trans-signalling,” that is, cell activation by the soluble IL-6/IL-6 receptor complex instead of more specific IL-6 binding to its surface receptor, is probably responsible for many detrimental effects of IL-6.^65,66^

The promoter polymorphisms of IL-6 have been repeatedly associated with different gene expression. The promoter region from −225 to −113, that is, involving the common −174 G/C polymorphism, contains regulatory elements which ensure transcription induction by IL-1 or TNFα.^67^ Indeed, following stimulation by IL-1, transcription is enhanced in the IL-6 −174 G-allele containing transfected HeLa cells when compared to the C-allele; this SNP can therefore be considered functional.^68^ The carriers of different genotypes have different plasmatic concentration of IL-6, which decreases in a sequence of GG > GC > CC in healthy people^68^ or in patients with brain vessel malformations,^69^ while CC carriers were shown to have higher IL-6 levels in days following coronary artery bypass graft surgery, suggesting a different pattern during inflammatory responses.^70^ This effect may be partially explained by linkage disequilibrium with another functional promoter SNP in the IL-6 gene, that is, −6331 T/C, located in the binding site for enhancing transcription factor Oct-1.^71^ Other polymorphisms in the IL-6 gene have also been shown to influence its expression in interaction with −174 G/C. They include −597 G/A, SNP in strong linkage disequilibrium with −174 G/C, which was confirmed in our study.^72,73^ Following the administration of lipopolysaccharides, bacterial surface molecules capable of inducing foam cell formation,^74^ a lower secretion of IL-6 was observed in subjects with −597 G to −174 G haplotypes compared to −597 A to −174 C haplotypes.^73^

The relationship between IL-6 polymorphisms and CAD has been investigated for more than 20 years and may provide insight into causality in the association between high IL-6 levels and CAD. In a recent extensive meta-analysis of 50 studies involving over 30,000 patients, no significant association between IL-6 −174 G/C and coronary artery disease onset was established in patients of Caucasian origin. However, there was substantial heterogeneity among the studies. In subgroup analysis the G allele was protective in studies where population-based control subjects were used and in non-Caucasian populations.^75^ Data regarding survival of patients with confirmed CAD are scarcer. In patients presenting with acute coronary syndrome, the G allele was found to have detrimental effects on 1-year survival.^76^ On the other hand, in another study including 218 patients with chronic CAD and renal failure, subjects carrying the G allele had a significantly better prognosis which was found to be consistent with our results.^77^ It is possible that mildly elevated IL-6 levels in −597 G to −174 G carriers could be beneficial due to their support of angiogenesis in chronic 3VD, while the increased risk of plaque destabilization is more important after acute coronary syndrome.

### Other Investigated Polymorphisms

In the present study, none of the remaining 21 investigated polymorphisms constituted significant independent risk factors of death in patients suffering from 3VD, although the association with CAD has been previously reported in many cases. For example, promoter polymorphisms of the MMP-2 gene −1306 C/T and −790 T/G have been associated with the presence of CAD in a meta-analysis of 9 studies^78^ including a study conducted by our research group which compared patients with 3VD to controls.^36^ The genetic variation of RAAS has also been extensively studied in cardiovascular research. Polymorphisms of ACE and angiotensinogen have been associated with different angiotensin II levels, as well as with cardiovascular risk.^79^ A common polymorphism in MTHFR, 677 C/T, has been associated with the risk of atherosclerosis in Asian populations. In Europeans, who have a relatively higher folate intake, no significant contribution of MTHFR 677 C/T to the onset of CAD has been established^80^; however, there might be greater risk for the T allele in otherwise risky subgroups.^81^ The intron polymorphism of the ET-1 gene 8002 G/A has been associated with myocardial infarction by our group.^33^ In our previous research we failed to prove the effect of polymorphisms in PPAR-α PPAR-γ and RXR-α on prognosis after PCI^32^; however, variation in the RXR-α intron was associated with all-cause heart failure.^82^ Furthermore, 161 C/T in PPAR-γ has been linked to CAD among the Chinese (but not Caucasians) in a recent meta-analysis.^83^

The APO E locus belongs to 35 loci which have been associated with CAD in GWAS.^8^ While ε4 carriers are at a slightly higher risk of atherosclerosis compared to most frequent ε3/ε3 homozygotes, the ε2 allele is protective in heterozygotes and ε2/ε2 homozygotes have a variable risk of atherosclerosis.^84^ With respect to tumour necrosis factors, association studies of TNF-α promoter variants have produced various results^85–87^ while the role of polymorphism B1/B2 (+252 G/A) in TNF-β, previously suggested as contributing to CAD onset, was found to be insignificant in a meta-analysis of 22 studies.^88^

Since data from other studies regarding the effect of the mentioned variants on CAD patient survival are largely missing, this study contributes to understanding their role in the progression of atherosclerosis. The long-term prospective approach should be a useful tool with respect to the evaluation of the contribution of these variants to the prognosis of patients suffering from severe CAD. In our study, only the genetic variation of the IL-6 promoter added new, independent information besides the characteristics of cardiac involvement and the traditional cardiovascular risk factors.

### Limitations

The study has several limitations. Firstly, all patients were enrolled in single institution, which limits the generalizability of our results. Other limitation is relatively low number of participants with all available data which reduces the power of the study; however the power is gained by relative homogeneity of the study group and long follow-up time. The power could have not been high enough to detect all potential genetic and non-genetic effects and larger multicentre studies are needed in this respect. The disappearance of the CABG effect in a multivariate model suggests the role of patient selection for the surgery; in subsequent analysis, patients with higher EF were more frequently selected for CABG, and EF was the most significant predictor of survival in stepwise model. However, the fact that CABG was not a significant independent predictor does not contradict the established therapeutic benefit of the procedure; it is rather a consequence of the small size of the PCI and pharmacological treatment groups, and therefore low power of the test.

## CONCLUSION

Age, increased BMI, diabetes, low ejection fraction, left main stenosis, and genetic variation in the IL-6 promoter were established as significant independent risk factors for the survival of patients with three-vessel disease. The G alleles of promoter polymorphisms rs1800795 (−174 G/C) and rs1800797 (−597 G/A) were associated with lower mortality.
